# Draft Genome Sequence of Serratia sp. Strain S1B, Isolated from Mercury-Contaminated Soil

**DOI:** 10.1128/genomeA.00534-18

**Published:** 2018-06-21

**Authors:** Ashish Pathak, Rajneesh Jaswal, Xiaoyu Xu, Ashvini Chauhan

**Affiliations:** a School of the Environment, Florida A&M University, Tallahassee, Florida, USA; b Savannah River Ecology Laboratory, Aiken, South Carolina, USA; c University of Georgia, Athens, Georgia, USA

## Abstract

We report here the draft genome sequence of Serratia sp. strain S1B, comprising 7,710,841 bases, 7,075 coding sequences, a G+C content of 45.9%, and 138 RNAs. Notably, a repertoire of biodegradative genes, several occurring on genomic islands, was also identified, which enhances our understanding of the environmental relevance of Serratia spp.

## GENOME ANNOUNCEMENT

The Savannah River Site (SRS), managed by the U.S. Department of Energy (DOE), is a former nuclear facility, where metal-clad uranium (U) targets were fabricated for plutonium production (1, 2). Microorganisms isolated from SRS soils can serve as models to understand genomic mechanisms underpinning metal-microbe interactions and, in particular, bioremediation mechanisms from long-term contamination exposure. Toward this end, several bacterial strains were isolated from soils of the Savannah River Swamp System (sample ID SRSS-1) soils. The SRSS site is located at the confluence of Four Mile Creek and the Savannah River, which received liquid mercury (Hg) effluents from a chloralkali facility near Augusta, Georgia, USA, until the 1980s (3, 4). Cores were shipped on ice to the Florida A&M laboratory, where sediments were serially diluted and plated onto LB media containing Hg (5 µg/ml, provided as mercuric chloride) to isolate Hg-resistant bacteria. One robustly growing strain, labeled as strain S1B, was selected for genomic studies.

Briefly, DNA from strain S1B was extracted and sequenced using an Illumina HiSeq 2000 instrument as described previously (5). Raw reads were *de novo* assembled with the SPAdes assembler (6) using default settings. Coverage statistics were computed by mapping raw reads to the assembled genome using Bowtie 2 (7); contigs with low coverage were removed. Genome island analysis was performed with Island Viewer (8), and taxonomic affiliation of strain B1 was assessed using the One Codex database.

Approximately 65.36% of the genomic reads from strain S1B were taxonomically affiliated with Serratia sp. DD3, but 16S rRNA gene PCR sequencing suggested closer affiliation with Serratia sp. S_T_TSA72. The genome sequence, with *N*_50_ and *L*_50_ values of 108,969 and 17 bp, respectively, was assembled in 153 contigs with a coverage of 300×. Annotation was performed with the Rapid Annotations using Subsystems Technology (RAST) server (9) (https://doi.org/10.6084/m9.figshare.6253850), which revealed the genomic size of approximately 7,710,841 bases (https://doi.org/10.6084/m9.figshare.6253847.v2) with 7,075 coding sequences and a GC content of 45.9%. A total of 585 subsystems were annotated from strain S1B with genes (number in parentheses) for membrane transport (282), stress response (221), metabolism of aromatic compounds (173), motility and chemotaxis (9), among others. Several gene homologs, previously shown to be involved in the resistance against heavy metals/radionuclides, including the cobalt-zinc-cadmium efflux system, membrane transporters, and antimicrobial extrusion proteins, were also identified, which likely facilitates the survival of strain S1B in an Hg-contaminated habitat.

Notable evolutionary traits in strain S1B included the presence of several genomic islands (GEIs) (https://doi.org/10.6084/m9.figshare.6253853), which provide genomic plasticity and beneficial features to the host (10). Specifically, relative to the genome of the metabolically versatile strain S. marcescens Db11, strain S1B contained a suite of interspersed genes coding for biodegradation and metal homeostasis. The latter aids cellular uptake, efflux, and sensory pathways maintaining low intracellular concentration of metals. Taken together, our findings show that the metal-resisitant Serratia sp. strain S1B harbors a variety of metal-resistant genes, many linked to GEIs. Such genome-enabled studies not only add to our understanding of metal-microbe interactions but will also potentially result in the identification of genomic markers to study bioremediation efficiency and restoration success of the historically contaminated SRS habitats.

### Accession number(s).

This whole-genome shotgun project of strain S1B has been deposited at DDBJ/ENA/GenBank under the accession number PYUJ00000000.
